# Real-world experience of erenumab in patients with chronic or episodic migraine in the UAE

**DOI:** 10.1186/s12883-022-02710-5

**Published:** 2022-06-16

**Authors:** Taoufik Alsaadi, Suzan Noori, Razmig Varakian, Saly Youssef, AbuBaker Almadani

**Affiliations:** 1 Department of Neurology, American Center of Psychiatry and Neurology, Abu Dhabi, United Arab Emirates; 2 University of Sharjah, Sharjah, United Arab Emirates; 3 Representative office, Novartis Middle East FZE, Dubai, United Arab Emirates; 4 Neurology Department, Rashid Hospital, Dubai, United Arab Emirates

**Keywords:** Erenumab, Calcitonin gene-related peptide monoclonal antibody, Migraine, Safety, Real-world evidence, UAE, CGRP

## Abstract

**Background:**

Erenumab is a fully human monoclonal antibody and a highly potent, first-in-class calcitonin gene-related peptide receptor inhibitor approved for migraine prevention in adults. Randomised, placebo-controlled trials show that erenumab treatment results in clinically meaningful responses, including significant reductions in monthly migraine days. Real-world evidence of the effectiveness of erenumab in patients with migraine is accruing, but gaps remain, and findings may vary according to region. We evaluated the usage patterns and effectiveness of erenumab in real-world settings in patients with migraine in the United Arab Emirates (UAE).

**Methods:**

This retrospective, observational real-world study enrolled patients ≥ 18 years with migraine who were prescribed erenumab in the UAE. Data were collected at baseline and Months 1, 3 and 6. The primary study objective was to characterise usage patterns of erenumab in patients with chronic migraine (CM) or episodic migraine (EM) in real-world settings in the UAE.

**Results:**

Of the 166 patients, 124 (74.7%) were females. The mean (standard deviation) age at migraine onset was 29 (7.93) years. Seventy-one patients (42.8%) had CM and 95 (57.2%) had EM.

In the overall population, the mean monthly headache/migraine days (MHD) at baseline was 15.7 (8.45) and mean change from baseline was − 8.2 (8.83) at Month 1, − 11.0 (9.15) at Month 3 and − 11.3 (8.90) at Month 6. The mean change from baseline in monthly acute migraine-specific medication days (MSMD) was − 9.0 (8.07) at Month 1, − 9.7 (8.73) at Month 3 and − 10.7 (8.95) at Month 6. At all time points, most patients achieved at least 50% reduction in MHD (80%–91%) and MSMD (84%–94%).

Similar reductions in MHD and MSMD and clinical benefit in CM or EM were seen with erenumab monotherapy or erenumab add-on therapy, with or without dose escalation and for treatment naïve or ≥ 1 previous preventive treatment failures, with additional clinical benefit in the erenumab add-on therapy and dose escalation to 140 mg subgroups.

**Conclusion:**

In this real-world study on erenumab use in the UAE, patients prescribed erenumab achieved clinically meaningful reductions in MHD and MSMD at all assessed time points. Erenumab was well tolerated with no new safety events.

**Supplementary Information:**

The online version contains supplementary material available at 10.1186/s12883-022-02710-5.

## Background

Migraine is a common chronic neurovascular disease associated with a significant personal burden of pain, disability, and reduced quality of life. The personal, social, and healthcare-related economic burden associated with the disease is also significant [1, 2]. Migraine remains under-recognised and undertreated [1]. Despite being under-recognised, the Global Burden of Disease 2016 study suggests a global age-standardised migraine prevalence of 14.3% [3]. The disease represents the second-largest cause of disability globally, with women having a higher number of years lived with disability (YLD) than men [4].

Headache disorders are the third-highest cause of YLD in the Middle East [4]. The prevalence of migraine in Arab countries is similar to that estimated worldwide but varies by region. The prevalence of migraine is reported to be 2.6%–5% in Saudi Arabia and 7.9% in Qatar, whereas the 1-year migraine prevalence in Oman is reported to be 10.1% [5] and in Kuwait is reported to be 23% [6].

Preventive treatment of migraine is typically based on pharmacological therapies such as topiramate, valproate, β-blockers and amitriptyline, which were not developed specifically for migraine management. These non-specific therapies have been associated with high levels of discontinuation owing to a lack of efficacy and/or poor tolerability [7]. Calcitonin gene-related peptide (CGRP) plays a key role in migraine pathophysiology [8]; hence, the CGRP receptor is a relevant target for the preventive treatment of migraine [9, 10].

Erenumab, a fully human immunoglobulin G2 monoclonal antibody (mAb), is a highly potent and selective antagonist of the canonical CGRP receptor [11]. Erenumab was specifically developed to treat migraine [11] and is a first-in-class mAb approved by the United States Food and Drug Administration (May 2018) and the European Medicines Agency (July 2018) for migraine prevention in adults [12, 13]. Erenumab has since been approved in several other countries, including the United Arab Emirates (UAE), and is administered as monthly subcutaneous injections at a dose of either 70 mg or 140 mg [12]. In the UAE, the recommended starting dose is 70 mg, which can be escalated to 140 mg if required.

Erenumab has been previously shown to reduce the monthly migraine days (MMD) and increase the likelihood of achieving a clinically meaningful response (≥ 50% reduction from baseline in MMD) at all monthly assessment points tested in placebo-controlled trials, including pivotal phase 2/3 trials of episodic migraine (EM) [14–16] and chronic migraine (CM) [17]. There is a need to understand the region-specific usage patterns and effectiveness of erenumab. This study aimed to characterise the real-world usage patterns and effectiveness of erenumab in the UAE.

## Methods

### Study design

This was a retrospective, non-interventional, observational, real-world study that enrolled patients diagnosed with either EM (up to 14 monthly headache/migraine days [MHD] [18]) or CM (≥ 15 MHD, of which at least 8 had to be typical migraine days), including patients with medication overuse headache (MOH) treated with erenumab at one of the four sites across three medical centres in the UAE. MOH was defined according to the International Classification of Headache Disorders, 3^rd^ edition guidelines [19].

Patients aged ≥ 18 years at the time of erenumab treatment initiation who had received at least three doses of erenumab before entering the study were included in the analysis. Patients aged ≥ 50 years at migraine onset were excluded from this analysis. Patients who were previously treatment naïve (i.e., for whom erenumab was prescribed as the first preventive therapy) and those who had ≥ 1 previous preventive treatment failure (PPTF) were included. Patients for whom erenumab was prescribed as the first preventive therapy had to have received at least 3 doses of erenumab before entering the study. For patients entering the study on erenumab 70 mg, the dose could be escalated to 140 mg at any time point following an initial three-month (every 28 day) administration of 70 mg upon the treating physician’s decision.

Demographics, baseline information, clinical characteristics and treatment history were extracted from the medical records of eligible patients. Data were collected at baseline, and at Months 1, 3 and 6, per availability in the medical records. Patient records with at least 6 months of follow-up from the time of erenumab treatment initiation were included in the analysis. The collected data are presented by migraine type (EM or CM); by erenumab monotherapy (with or without a sufficient washout period from the previous preventive treatments) or erenumab add-on therapy; by patients who entered the study and remained on erenumab 70 mg or those who were prescribed erenumab at the 70-mg dose and were subsequently escalated to erenumab 140 mg; and by treatment naïve or with PPTFs.

All relevant ethical, health authority, data privacy and site-specific approvals were obtained prior to the start of the study. The study was conducted in accordance with the principles of the Declaration of Helsinki. Informed consent was obtained from patients before data collection. Depending on each site’s local Institutional Review Board (IRB) regulations, a waiver of informed consent from the corresponding ethics committee was obtained. All authors were given access to the study data.

### Objectives and endpoints

The primary objective of the study was to characterise the usage patterns of erenumab in patients with EM or CM starting treatment with erenumab in a real-world setting in the UAE. The secondary objectives of the study were to evaluate the change in MHD in patients with CM and EM and the change in monthly acute migraine-specific medication days (MSMD), and to summarise patient demographic characteristics and disease/medication history.

Exploratory objectives included a description of compliance and adherence to erenumab, the response rate in terms of reduction in MHD across the study period and the incremental benefit of dose escalation from 70 to 140 mg. The enrolled analysis set included all patients who signed an informed consent form (ICF) or a waiver of ICF (wherever applicable) and met the inclusion and exclusion criteria. Male and female patients ≥ 18 years at the time of erenumab treatment initiation and with a diagnosis of EM or CM, including those with MOH, and treated with at least three doses of erenumab were enrolled. Patients > 50 years of age at migraine onset were excluded. The full analysis set (FAS) included all enrolled patients who received at least one dose of the study drug and had at least one evaluable post-baseline assessment. The safety analysis set included all patients who received at least one dose of the study drug.

Current disease status, including the duration of disease, the number of MHD, previous and current preventive treatments and comorbidities (anxiety, depression, diabetes, or obesity) were assessed at baseline. Medical history/current medical conditions and adverse events (AEs) were coded using the Medical Dictionary for Regulatory Activities.

### Statistical analysis

The confidence interval method for paired mean difference was used to estimate the sample size. The 95% CI was used. There was no power. Sample size calculations indicated that a minimum of 160 patients were required to characterise the effectiveness of erenumab in patients with CM or EM. Given the exploratory nature of the endpoints, descriptive statistics are presented. No formal hypotheses were defined for the present exploratory analyses; therefore, no inferential testing was performed on any endpoint. Categorical data are reported as frequencies and percentages, and continuous data as mean, standard deviation (SD), median, minimum, and maximum. *P*—values were derived from Chi-Square test or exact test for categorical variables and from t-test or Wilcoxon test for continuous variables. Owing to the descriptive and observatory nature of this study, missing data were not imputed.

## Results

### Demographics and baseline characteristics

Overall, 166 patients were enrolled and completed the study; 135 patients (81.3%) completed the study per protocol from baseline to Month 6, and 31 patients (18.7%) completed the study protocol partially. Of the 166 patients, a majority (*n* = 124, 74.7%) were female (Table 1). Seventy-one (42.8%) patients had CM and 95 (57.2%) had EM.

**Table 1:** Patient demographics and baseline characteristics

	**Overall *N* = 166**	**Chronic migraine *N* = 71**	**Episodic migraine *N* = 95**	***p*—value**
Age, years, mean (SD)	37.8 (8.99)	38.8 (9.51)	37.1 (8.57)	0.2502
Age categories, years, n (%)				
≥ 18 to < 35	65 (39.2)	24 (33.8)	41 (43.2)	0.0498
≥ 35 to < 50	89 (53.6)	38 (53.5)	51 (53.7)	
≥ 50	12 (7.2)	9 (12.7)	3 (3.2)	
Sex, female, n (%)	124 (74.7)	49 (69.0)	75 (78.9)	
Ethnicity, n (%)				
Local	123 (74.1)	53 (74.6)	70 (73.7)	0.8885
Non local	43 (25.9)	18 (25.4)	25 (26.3)	
Arab	27 (16.3)	12 (16.9)	15 (15.8)	
Asian	4 (2.4)	1 (1.4)	3 (3.2)	
Western	4 (2.4)	2 (2.8)	2 (2.1)	
European	7 (4.2)	3 (4.2)	4 (4.2)	
Other	1 (0.6)	0	1 (1.1)	
BMI, kg/m^2^, mean (SD)	28.2 (4.76)	28.4 (4.97)	28.1 (4.66)	0.8322
BMI category, kg/m^2^, n (%)				
≥ 18.5 to < 25	20 (26.0)	8 (25.8)	12 (26.1)	0.9985
≥ 25 to < 30	32 (41.6)	13 (41.9)	19 (41.3)	
> 30	25 (32.5)	10 (32.3)	15 (32.6)	
Family history of migraine, n (%)				
Yes	51 (30.7)	21 (29.6)	30 (31.6)	0.8952
No	76 (45.8)	34 (47.9)	42 (44.2)	
Unknown	39 (23.5)	16 (22.5)	23 (24.2)	
Duration of migraine, years, mean (SD)	5.0 (9.75)	6.0 (7.82)	4.4 (10.92)	0.0223
Age at migraine onset, years, mean (SD)	29.0 (7.93)	29.4 (8.35)	28.7 (7.63)	0.5352
MHD over the past 1 month, days, mean (SD)	15.6 (8.54)	22.9 (6.35)	9.0 (3.05)	< 0.0001
MHD at baseline, days, mean (SD)	15.7 (8.45)	22.9 (6.57)	9.2 (2.93)	NA
MSMD at baseline, days, mean (SD)	12.8 (10.00)	18.6 (8.55)	7.4 (8.11)	
Number of PPTFs, n (%)				
0	72 (43.4)	31 (43.7)	41 (43.2)	0.4886
1	28 (16.9)	14 (19.7)	14 (14.7)	
2	34 (20.5)	10 (14.1)	24 (25.3)	
3	5 (3.0)	2 (2.8)	3 (3.2)	
4	17 (10.2)	8 (11.3)	9 (9.5)	
≥ 5	10 (6.0)	6 (8.5)	4 (4.2)	
Number of classes of preventive therapy used previously, n (%)				
1	34 (20.5)	9 (12.7)	25 (26.3)	
2	10 (6.0)	2 (2.8)	8 (8.4)	
3	5 (3.0)	4 (5.6)	1 (1.1)	
> 3	4 (2.4)	2 (2.8)	2 (2.1)	

Percentages are based on the total number of subjects in the full analysis set. *P*—value is derived from a Chi-square test or exact test for categorical variables and from t-test or Wilcoxon test for continuous variables. BMI is calculated as follows: (body weight in kg)/ (height in m)^2^; percentages for BMI categories are based on the total number of patients who had BMI data in the full analysis set. *BMI*, body mass index; *MHD*, monthly headache/migraine days; *MSMD*, monthly acute migraine-specific medication days; *n*, number of patients; *N*, total number of patients; *NA*, not available; *PPTF*, previous preventive treatment failure; *SD*, standard deviation

The demographic and baseline characteristics by CM and EM are presented in Table 1. The overall mean (SD) age of patients was 37.8 (8.99) years (Table 1). The difference in mean age between patients with CM and EM was not statistically significant; however, for age categories, the difference was statistically significant (*p* = 0.0498). Most patients were aged ≥ 35 years to < 50 years (53.6%) and a similar proportion of those with CM and EM were in this category. A higher proportion of patients with EM tended to be in the ≥ 18 to < 35-year category and a lower proportion in the ≥ 50-year category compared with those with CM (EM: 43.2% and 3.2%; CM: 33.8% and 12.7%, respectively). A family history of migraine was reported for 30.7% of patients (Table 1). The mean (SD) age at onset of migraine for the overall study population was 29.0 (7.93) years, 29.4 (8.35) years for CM and 28.7 (7.63) years for EM (Table 1).

The mean (SD) number of MHD over one month prior to enrolment in the overall population was 15.6 (8.54) days and, as expected, was significantly higher with CM (22.9 [6.35] days) than with EM (9.0 [3.05] days; *p* < 0.0001). The mean (SD) number of MSMD at baseline was 12.8 (10.0) days, and this difference was statistically significant (*p* < 0.0001) between the CM (18.6 [8.55] days) and EM subgroups (7.4 [8.11] days).

Baseline demographics and characteristics of all subgroups are presented in Table S1. All patients started erenumab at the 70-mg dose. The number of patients on erenumab 70 mg throughout the study was 98 (59.0%) and the dose was escalated to 140 mg in 68 (41.0%) patients. Overall, 72 (43.4%) patients had no PPTFs prior to receiving erenumab, whereas 94 (56.6%) had ≥ 1 PPTF.

**Fig. 1 a:**
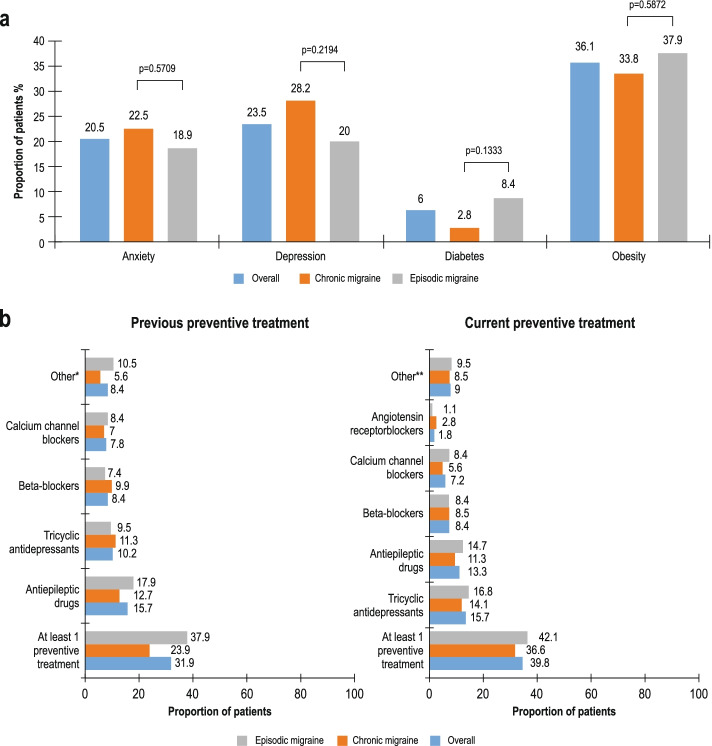
Proportion of patients with comorbidities – by overall population, migraine type and monotherapy and add-on therapy. **b **Proportion of patients with previous or current preventive treatment – Overall population and by migraine type. *Botulinum toxin type A, antiepileptics, antimigraine preparations, flunarizine dihydrochloride, galcanezumab, local anaesthetics. **Magnesium oxide, botulinum toxin type A, antiepileptics, antimigraine preparations, local anaesthetics, magnesium, venlafaxine. Percentages are based on the total number of subjects in the full analysis set

No statistically significant differences were observed in the proportion of patients with CM and EM who had comorbidities (Fig. 1a). Comorbidities by subgroup are presented in Fig. S1. In total, 53 patients (31.9%) received at least one previous preventive treatment (Fig. 1b). Antiepileptics (15.7%), tricyclic antidepressants (10.2%), β-blockers (8.4%) and calcium channel blockers (7.8%) were the most frequently received previous preventive treatments (> 5% of patients). In this study, 100 (60.2%) patients received erenumab as monotherapy, whereas 66 (39.8%) received erenumab as an add-on to a current preventive therapy (Fig. 1b). Tricyclic antidepressants (15.7%), antiepileptics (13.3%), β-blockers (8.4%) and calcium channel blockers (7.2%) were the most frequently received current preventive treatments (> 5% of patients). There was a statistically significant difference between the proportions of patients receiving erenumab as monotherapy or add-on therapy (*p* = 0.0466), with higher rates of PPTFs in the add-on subgroup.

### Monthly headache/migraine days

**Fig. 2:**
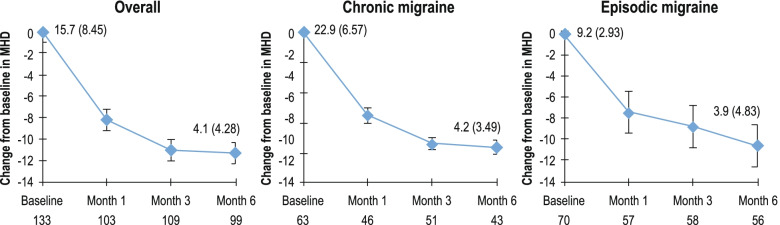
Change from baseline in MHD – Overall population and by migraine type. MHD, monthly headache/migraine days; SD, standard deviation. Mean ± standard error values are plotted and baseline MHD (SD) are provided for each plot. The total number of evaluable subjects at the respective visit in the full analysis set are presented below each plot. Baseline is defined as the last observation on the day of or before the first dose of the study drug. After baseline, only patients with a value at both baseline and the respective month are included

In the overall population, the mean (SD) number of MHD decreased from 15.7 (8.45) at baseline to 7.3 (7.54) at Month 1. A further decrease was noted at Month 3 (4.6 [4.83]) and Month 6 (4.1 [4.28]). The corresponding mean changes from baseline were − 8.2 (8.83) at Month 1, − 11.0 (9.15) at Month 3 and − 11.3 (8.90) at Month 6 (Fig. 2). A reduction in MHD was observed from baseline to Months 1, 3 and 6 in both CM and EM groups (Fig. 2).

**Fig. 3:**
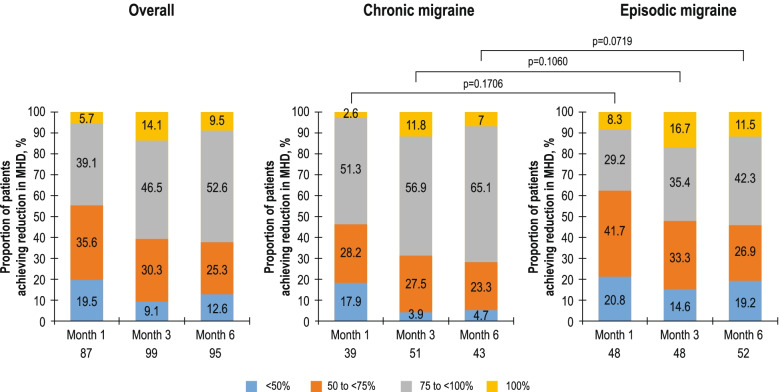
Proportion of patients achieving < 50%, 50% to < 75%, 75% to < 100% and 100% reduction in MHD over time. MHD, monthly headache days. Percentages are based on the total number of evaluable subjects at the respective visit in the full analysis set

At all time points, most patients in the overall population achieved at least a 50% reduction from baseline in MHD (80%–91%), and the proportion achieving the 75 to < 100% or 100% reduction categories was 39.1% and 5.7% at Month 1; 46.5% and 14.1% at Month 3; and 52.6% and 9.5% at Month 6, respectively (Fig. 3). The mean (SD) number of MHD at baseline was 16.9 (8.83) in the erenumab monotherapy subgroup and 13.9 (7.57) in the erenumab add-on therapy subgroup. A greater reduction in the number of MHD was observed with erenumab monotherapy compared with erenumab as add-on therapy at all assessed time points, but the differences were not statistically significant (*p* > 0.05 for all time points; Fig. S2). A similar trend in the reduction of mean MHD was observed in the subgroups of patients who were prescribed and remained on erenumab 70 mg and with dose escalation to 140 mg, as well as those in the treatment naïve and PPTF subgroups (Fig. S2). At all time points, most patients in the subgroup categories of with/without dose escalation, monotherapy/add-on therapy and treatment naïve/PPTF achieved at least a 50% reduction from baseline in MHD. As anticipated, at Month 1, a higher proportion of patients in the PPTF subgroup had a reduction from baseline in MHD by < 50% versus those who were treatment naïve; however, this proportion improved over time with erenumab treatment (Fig. S3).

### Monthly acute migraine-specific medication days

**Fig. 4:**
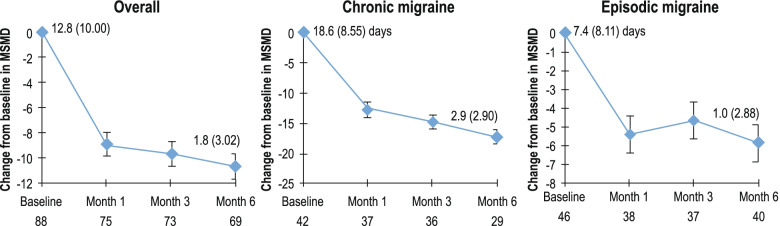
Change from baseline in MSMD – Overall population and by migraine type. MSMD, monthly acute migraine-specific medication days; SD, standard deviation. Mean ± standard error values are plotted and baseline MSMD (SD) are provided for each plot. Baseline is defined as the last observation on the day of or before the first dose of the study drug. After baseline, only patients with a value at both baseline and the respective month are included

At baseline, MSMD data were available for 88 patients. The mean (SD) MSMD at baseline in the overall population was 12.8 (10.00) and decreased to 4.1 (5.57) at Month 1, 2.9 (4.23) at Month 3 and 1.8 (3.02) at Month 6. The mean monthly change from baseline in MSMD was − 9.0 (8.07) at Month 1 and was reduced further to − 9.7 (8.73) at Month 3 and − 10.7 (8.95) at Month 6 (Fig. 4). Similar to that in the overall population, there was a decrease in mean MSMD from baseline to Month 1, Month 3 and Month 6 in both CM and EM groups (Fig. 4).

**Fig. 5:**
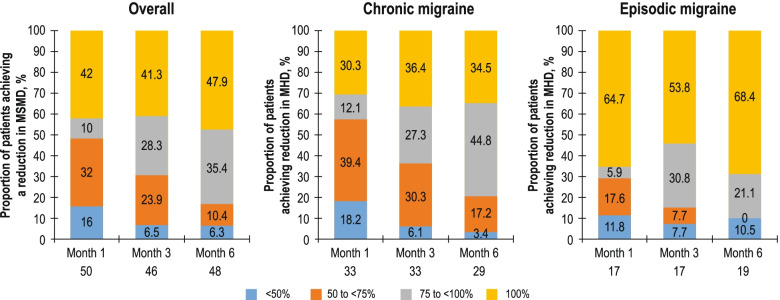
Proportion of patients achieving < 50%, 50% to < 75%, 75% to < 100% and 100% reduction in MSMD. MSMD, monthly acute migraine-specific medication days. Percentages are based on the total number of evaluable patients at the respective visit in the full analysis set

Similar to the reduction in MHD, most patients in the overall population and CM and EM groups achieved at least a 50% reduction from baseline in MSMD at all time points, and more than half of the patients in the EM group achieved a 100% reduction in MSMD at all time points (Fig. 5). In the subgroups of patients with and without dose escalation, the mean monthly change from baseline in MSMD at Month 1 was –10.8 (9.61) and –8.1 (6.98), respectively; for patients who were treatment naïve and who had PPTF, the means changes were –8.9 (7.74) and –9.1 (8.32), respectively; and for those receiving erenumab as monotherapy or add-on therapy, the mean changes were –10.3 (8.25) and –7.7 (7.77), respectively (Fig. S4). In the subgroup of patients escalated to the 140-mg dose, a majority achieved at least a 50% reduction from baseline at all time points, and the proportion of patients achieving either a 75% to < 100% or 100% reduction in MSMD increased from 76.5% at Month 3 (before dose escalation) to 84.2% at Month 6. The corresponding results in patients who remained on the 70-mg dose were 54.9% at Month 1, 65.5% at Month 3 and 82.7% at Month 6 (Fig. S5). A majority of patients achieved at least a 50% reduction at all time points in the monotherapy and add-on therapy subgroups and in the treatment naïve and PPTF subgroups. More than 35% of patients in the treatment naïve and PPTF subgroups had a 100% reduction in MSMD at all time points. Among those in the add-on therapy subgroup, 64% had a 100% reduction in MSMD by Month 6, increasing from 47.6% at Month 1 and 45% at Month 3.

### Medication overuse

There was a decrease in the number of patients in the overall population with overuse of at least one medication for ≥ 15 days per month from 49 (29.5%) at baseline to 12 (7.2%), 6 (3.6%) and 0 at Months 1, 3 and 6, respectively. Non-steroidal anti-inflammatory drugs were the most frequently used medications in the overall population at baseline (29 [17.5%]), which declined to 4 (2.4%) and 1 (0.6%) at Months 1 and 3, respectively.

### Exposure and safety

A total of 167 patients were included in the safety set (one patient who met the exclusion criteria was on medication and was excluded from the FAS but was included in the safety set). The mean (SD) treatment duration and on-treatment exposure were 5.6 (1.54) and 6.6 (1.54) months, respectively. Of the 167 patients, 34 (20.4%) experienced at least one AE. The most commonly reported AEs (> 1%) were constipation (3%), insomnia (2.4%) and influenza (1.8%) followed by falls, dizziness, arthralgia, stress and headache (1.2% each; Table 2). A majority (76.5%) of AEs were mild to moderate in severity. Two patients (1.2%) had severe AEs: atypical pneumonia and spontaneous abortion in one (0.6%) patient each. In the remaining 4.8% of patients, the AE severity was unknown. Five patients (3.0%) experienced at least one AE that was related to study treatment. All five patients experienced constipation (3 [1.8%] experienced mild AEs and the severity was unknown in 2 [1.2%]). Approximately 41% of patients (68 of 167) had a dose change (all of these were dose escalation from 70 to 140 mg upon the treating physician’s decision) during the study, but only three patients (1.8%) experienced AEs leading to dose adjustments/interruptions. No deaths were reported.

**Table 2:** Treatment-emergent AEs (> 1% of patients) by preferred term and maximum severity (safety set)

Preferred term	Mild	Moderate	Severe	Missing	Overall *N* = 167
Number of patients with at least 1 AE, n (%)	20 (12.0)	6 (3.6)	2 (1.2)	8 (4.8)	34 (20.4)
Constipation	3 (1.8)	0	0	2 (1.2)	5 (3.0)
Insomnia	4 (2.4)	0	0	0	4 (2.4)
Influenza	2 (1.2)	0	0	1 (0.6)	3 (1.8)
Fall	1 (0.6)	1 (0.6)	0	0	2 (1.2)
Arthralgia	0	1 (0.6)	0	1 (0.6)	2 (1.2)
Headache	2 (1.2)	0	0	0	2 (1.2)
Stress	2 (1.2)	0	0	0	2 (1.2)
Dizziness	1 (0.6)	0	0	1 (0.6)	2 (1.2)

Percentages are based on the total number of patients in the safety set. A patient with multiple AEs within a primary system organ class is counted only once in the total row. A patient with multiple occurrences of an AE under one treatment is counted only once in this AE category. *AE*, adverse event; *n*, number of patients; *N*, total number of patients

## Discussion

To the best of our knowledge, this is the first real-world study evaluating the experience of erenumab in patients with CM or EM in the UAE. A higher proportion of patients prescribed erenumab in the UAE were female, in the younger age categories, and more likely to have EM than CM [20]. The overall age distribution was older in the CM subgroup compared with the EM group, however, the mean age at onset of migraine was similar in both groups (approximately 29 years).

This study comprised both treatment-naïve patients (43%) and those who had ≥ 1 PPTF (57%). Most patients included in the study were prescribed erenumab as monotherapy; approximately 40% of patients received concomitant preventive therapy, implying that these patients represented a more difficult-to-treat population. For the overall population, a reduction in the number of MHD was observed at all assessed time points with a noticeable reduction at Month 1 and further reductions at Months 3 and 6. A similar trend was observed in the evaluation of MSMD. These findings were consistent with the results on change in MMD and MSMD from baseline observed in the global clinical trials of erenumab [14–17], as were the greater reductions in MHD and MSMD from baseline observed in patients with CM versus those with EM.

Our observations also accord with other studies of erenumab in the real world across a range of regions and settings [21–25]. For example, The TELESCOPE study, a multicentre survey collecting real-world data from 45 German headache centres, reported a 7.5 days reduction (*p* < 0.0001) in MHD following 3 months treatment with erenumab [25]. In another multicentre study in Italy (EARLY2), erenumab treatment reduced MHD by 12.8 ± 8.9 from baseline through week 45 to 48 [21].

Concordant changes from baseline in MHD and MSMD were observed across the other subgroups that were studied (with and without dose escalation, treatment naïve and PPTF, monotherapy and add-on therapy). Of note, a greater reduction in MHD was observed in patients initiating erenumab as monotherapy compared with those initiating erenumab as add-on therapy, although this difference was not statistically significant. Patients receiving erenumab as add-on therapy may comprise a more difficult-to-treat population, but a reduction in MHD and MSMD was still observed. Our results support the ability of erenumab to provide additional clinical benefits when administered concomitantly with other forms of preventive therapy.

In the overall population and across all subgroups, most patients achieved a ≥ 50% reduction in MHD and MSMD at all assessed time points and a substantial proportion of patients achieved 75 to < 100% and 100% reductions in MHD and MSMD during the study. Notably, this pattern of response was consistent in the subgroup of patients with dose escalation to 140 mg. This is in line with the erenumab prescribing information, which states that some patients can benefit from a dose increase from 70 to 140 mg [12]. Similar to those observed in global clinical studies, reductions from baseline in MHD and MSMD were observed in both the treatment naïve and PPTF subgroups, with most patients in these subgroups achieving a ≥ 50% reduction from baseline in MHD and MSMD [26, 27]. However, there was a significant difference at Month 1 in the proportion of patients achieving a ≥ 50% reduction from baseline in MHD in the treatment naïve versus PPTF subgroups.

In addition to the effects on MSMD, erenumab treatment was also associated with a decrease in the number of patients with medication overuse from 29.5% at baseline to 3.6% at month 3. This association was also observed in a prospective, single centre real-world audit from United Kingdom (*N* = 162), in which the number of patients with medication overuse decreased from 54% at baseline to 25% at Month 6 [24].

It is noteworthy that a higher proportion of patients than in the global clinical studies reached a response threshold of 100% reduction in MHD. This may be due to ethnic and regional differences of the population studied but may also be due to a higher expectation of disease improvement when using of a therapy known to have been beneficial in multiple other studies. Additionally, considering the entry requirement criterion of 3 initial injections of erenumab and 6 months of follow-up, this could have resulted in the higher 100% responder rates than in the pivotal trials.

The safety profile reported here is similar to that reported in previous studies and no new safety signals were reported. Most AEs were mild to moderate in nature. Constipation was the most frequently reported AE. Interestingly, the overall proportion of patients reporting any AE (20%) was lower in this study compared with that in global phase 2 and 3 studies (44%–57%) that evaluated erenumab 70 mg or 140 mg for 3 or 6 months in patients with CM and/or EM [14–17, 28]. There were no study or treatment discontinuations in this study, a finding that supports the favourable tolerability of erenumab.

The major limitations of this study are similar to those of other retrospectively designed studies, such as recall bias. By requiring patients to have taken at least 3 doses of erenumab for inclusion, the study may have excluded patients who discontinued treatment due to lack of efficacy or other reasons and may have skewed the results favourably in comparison to the pivotal clinical trial data. Headache is only one of the symptoms of migraine, and the selection of MHD instead of MMD as a secondary endpoint could be another limitation of this study. Additionally, headache and migraine days were not assessed separately, and headache type was abstracted only from information available in patient charts, which may lack granularity or consistency across centres [18].

## Conclusions

In this real-world study of the use of erenumab in the UAE, patients who were prescribed erenumab were more likely to have EM than CM. Most patients received erenumab as monotherapy and approximately 57% had ≥ 1 PPTF.

Irrespective of the demographics or baseline disease burden, clinically meaningful reductions in MHD and MSMD were observed at all assessed time points. In line with the higher baseline disease burden, the reduction in MHD and MSMD was greater in CM than EM. An additional clinical benefit was observed when erenumab was prescribed as add-on therapy and when its dose was increased from 70 to 140 mg by physicians. This is in line with the consistent numerical advantage of the 140-mg dose over the 70-mg dose observed on a range of endpoints in erenumab phase 2/3 trials [29].

Overall, the results presented here from the UAE are consistent with the data generated in global clinical trial programmes [14–17]. No new safety events were observed. The AEs observed in this study were consistent with the established safety profile of erenumab. These real-world results from the UAE are representative of the patient population with migraine, which includes both treatment-naïve patients and those who had PPTFs; therefore, these findings could be applied to routine clinical practice.

These real-world data further extend the evidence on the clinically meaningful effectiveness of erenumab to the population of patients living with migraine in the UAE.

## Supplementary information

**Additional file 1:Fig. S1. **Proportion of patients with comorbidities – with and without dose escalation, by treatment-naïve and failure and by monotherapy and add-on therapy. **Fig. S2. **Change from baseline in MHD – with and without dose escalation, by treatment naïve and failure and by monotherapy and add-on therapy. **Fig. S3.** Proportion of patients achieving <50%, 50% to <75%, 75% to <100% and 100% reduction in MHD over time – with and without dose escalation, by treatment naïve and failure and by monotherapy and add-on therapy. **Fig. S4. **Change from baseline in MSMD – with and without dose escalation, by treatment naïve and failure and by monotherapy and add-on therapy. **Fig. S5.** Proportion of patients achieving <50%, 50% to <75%, 75% to <100% and 100% reduction in MSMD over time – with and without dose escalation, by treatment naïve and failure and by monotherapy and add-on therapy. **Table S1.** Patient demographics and baseline characteristics.

## Data Availability

The data that support the findings of this study are available from [Novartis Middle East FZE] but restrictions apply to the availability of these data, which were used under license for the current study, and so are not publicly available. Data are however available from the authors upon reasonable request and with permission of [Novartis Middle East FZE].

## Abbreviations

AE: Adverse event
BMI: Body mass index
CGRP: Calcitonin gene-related peptide
CM: Chronic migraine
EM: Episodic migraine
FAS: Full analysis set
ICF: Informed consent form
IRB: Institutional Review Board
MHD: Monthly headache/migraine days
MMD: Monthly migraine days
MOH: Medication overuse headache
MSMD: Migraine-specific medication days
PPTF: Previous preventive treatment failure
SD: Standard deviation
UAE: United Arab Emirates
YLD: Years lived with disability
