# Supplementary material for: Real-world experience of erenumab in patients with chronic or episodic migraine in the UAE
Source: BMC Neurol. 2022 Jun 16;22:221. doi: 10.1186/s12883-022-02710-5 (PMC9202108; doi:10.1186/s12883-022-02710-5)
Supplement: Supplementary file 1 — Additional file 1:Fig. S1. Proportion of patients with comorbidities – with and without dose escalation, by treatment-naïve and failure and by monotherapy and add-on therapy. Fig. S2. Change from baseline in MHD – with and without dose escalation, by treatment naïve and failure and by monotherapy and add-on therapy. Fig. S3. Proportion of patients achieving <50%, 50% to <75%, 75% to <100% and 100% reduction in MHD over time – with and without dose escalation, by treatment naïve and failure and by monotherapy and add-on therapy. Fig. S4. Change from baseline in MSMD – with and without dose escalation, by treatment naïve and failure and by monotherapy and add-on therapy. Fig. S5. Proportion of patients achieving <50%, 50% to <75%, 75% to <100% and 100% reduction in MSMD over time – with and without dose escalation, by treatment naïve and failure and by monotherapy and add-on therapy. Table S1. Patient demographics and baseline characteristics. [file 12883_2022_2710_MOESM1_ESM.pdf]

Table S1 Patient demographics and baseline characteristics

	Without dose escalation N=98	With dose escalation N=68	p-value	Treatment naïve N=72	PPTF N=94	p-value	Monotherapy N=100	Add-on therapy N=66	p-value
Age, years, mean (SD)	37.4 (9.02)	38.5 (8.97)	0.4047	35.8 (7.54)	39.4 (9.73)	0.0093	37.6 (8.85)	38.3 (9.26)	0.6250
Age categories, years, n (%)									
≥18 to <35	39 (39.8)	26 (38.2)	0.4447	35 (48.6)	30 (31.9)	0.0100	41 (41.0)	24 (36.4)	0.3824
≥35 to <50	54 (55.1)	35 (51.5)		36 (50.0)	53 (56.4)		50 (50.0)	39 (59.1)	
≥50	5 (5.1)	7 (10.3)		1 (1.4)	11 (11.7)		9 (9.0)	3 (4.5)	
Sex, female, n (%)	72 (73.5)	52 (76.5)	0.6618	57 (79.2)	67 (71.3)	0.2465	73 (73.0)	51 (77.3)	0.5354
Ethnicity, n (%)									
Local	71 (72.4)	52 (76.5)	0.5608	54 (75.0)	69 (73.4)	0.8161	69 (69.0)	54 (81.8)	0.0651
Non local	27 (27.6)	16 (23.5)		18 (25.0)	25 (26.6)		31 (31.0)	12 (18.2)	
Arab	18 (18.4)	9 (13.2)		14 (19.4)	13 (13.8)		18 (18.0)	9 (13.6)	
Asian	2 (2.0)	2 (2.9)		0	4 (4.3)		3 (3.0)	1 (1.5)	
Western	4 (4.1)	0		2 (2.8)	2 (2.1)		3 (3.0)	1 (1.5)	
European	2 (2.0)	5 (7.4)		2 (2.8)	5 (5.3)		6 (6.0)	1 (1.5)	
Other	1 (1.0)	0		0	1 (1.1)		1 (1.0)	0	
BMI, kg/m^2, mean (SD)	28.2 (4.81)	28.3 (4.71)	0.7406	28.6 (5.69)	28.0 (4.10)	0.7382	27.8 (5.44)	28.6 (4.09)	0.4228
BMI category, kg/m^2, n (%)									
≥18.5 to <25	16 (27.1)	4 (22.2)	0.9435	10 (33.3)	10 (21.3)	0.4647	13 (36.1)	7 (17.1)	0.1640
≥25 to <30	24 (40.7)	8 (44.4)		12 (40.0)	20 (42.6)		13 (36.1)	19 (46.3)	
>30	19 (32.2)	6 (33.3)		8 (26.7)	17 (36.2)		10 (27.8)	15 (36.6)	
Family history of migraine, n (%)									
Yes	28 (28.6)	23 (33.8)	0.0063	20 (27.8)	31 (33.0)	0.4961	33 (33.0)	18 (27.3)	0.0771
No	54 (55.1)	22 (32.4)		32 (44.4)	44 (46.8)		39 (39.0)	37 (56.1)	
Unknown	16 (16.3)	23 (33.8)		20 (27.8)	19 (20.2)		28 (28.0)	11 (16.7)	
Duration of migraine, years, mean (SD)	4.9 (8.66)	5.4 (11.53)	0.2032	4.6 (11.65)	5.5 (7.61)	0.0020	4.8 (10.57)	5.5 (8.28)	0.2415
Age at migraine onset, years, mean (SD)	28.5 (7.06)	29.7 (9.03)	0.3815	29.5 (7.56)	28.7 (8.22)	0.5290	29.2 (8.69)	28.7 (6.71)	0.7095
MHD over the past 1 month, days, mean (SD)	15.3 (8.44)	16.1 (8.74)	0.6087	14.6 (8.48)	16.5 (8.54)	0.1933	16.6 (8.92)	14.2 (7.81)	0.1670
MHD at baseline, days, mean (SD)	NA	NA		NA	NA		16.9 (8.83)	13.9 (7.57)	NA
MSMD at baseline, days, mean (SD)	NA	NA		NA	NA		13.8 (10.19)	11.8 (9.85)	
Number of drug classes of preventive therapy used previously, n (%)									
1	22 (22.4)	12 (17.6)		1 (1.4)	33 (35.1)		17 (17.0)	17 (25.8)	
2	6 (6.1)	4 (5.9)		0	10 (10.6)		3 (3.0)	7 (10.6)	
3	4 (4.1)	1 (1.5)		0	5 (5.3)		3 (3.0)	2 (3.0)	
>3	1 (1.0)	3 (4.4)		0	4 (4.3)		4 (4.0)	0	

BMI, body mass index; MHD, monthly headache days; MSMD, monthly acute migraine-specific medication days; n, number of patients; N, total number of patients; NA, not available; SD, standard deviation

Percentages are based on the total number of subjects in the full analysis set. P-value is derived from a Chi-square test or exact test for categorical variable and from t-test or Wilcoxon test for continuous variable.

BMI is calculated as: (body weight in kg)/ (height in m)^2; percentages for the BMI categories are based on the total number of patients who have BMI in the full analysis set.

Fig. S1 Proportion of patients with comorbidities – with and without dose escalation, by treatment-naïve and failure and by monotherapy and add-on therapy

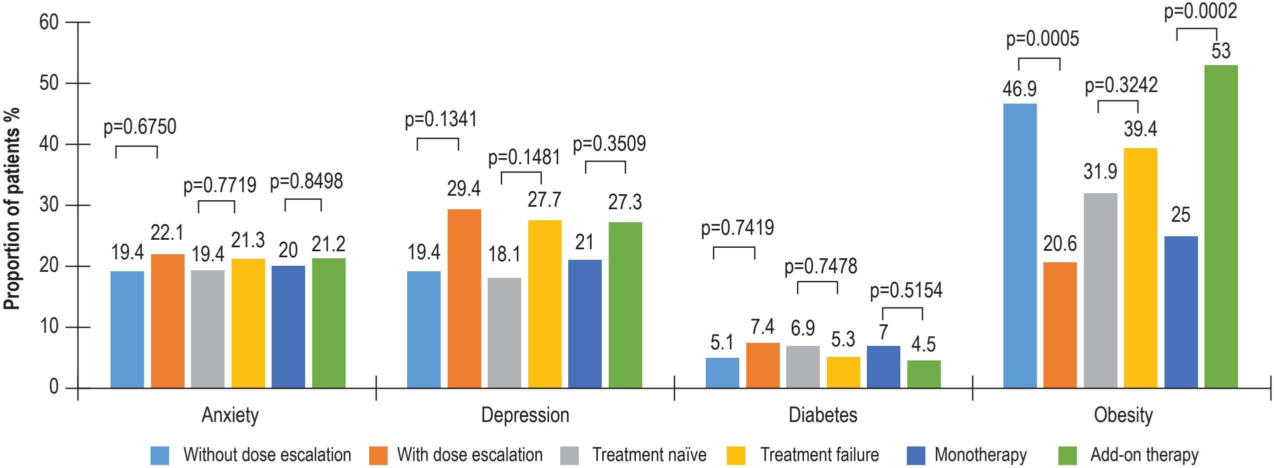

Fig. S2 Change from baseline in MHD – with and without dose escalation, by treatment naïve and failure and by monotherapy and add-on therapy

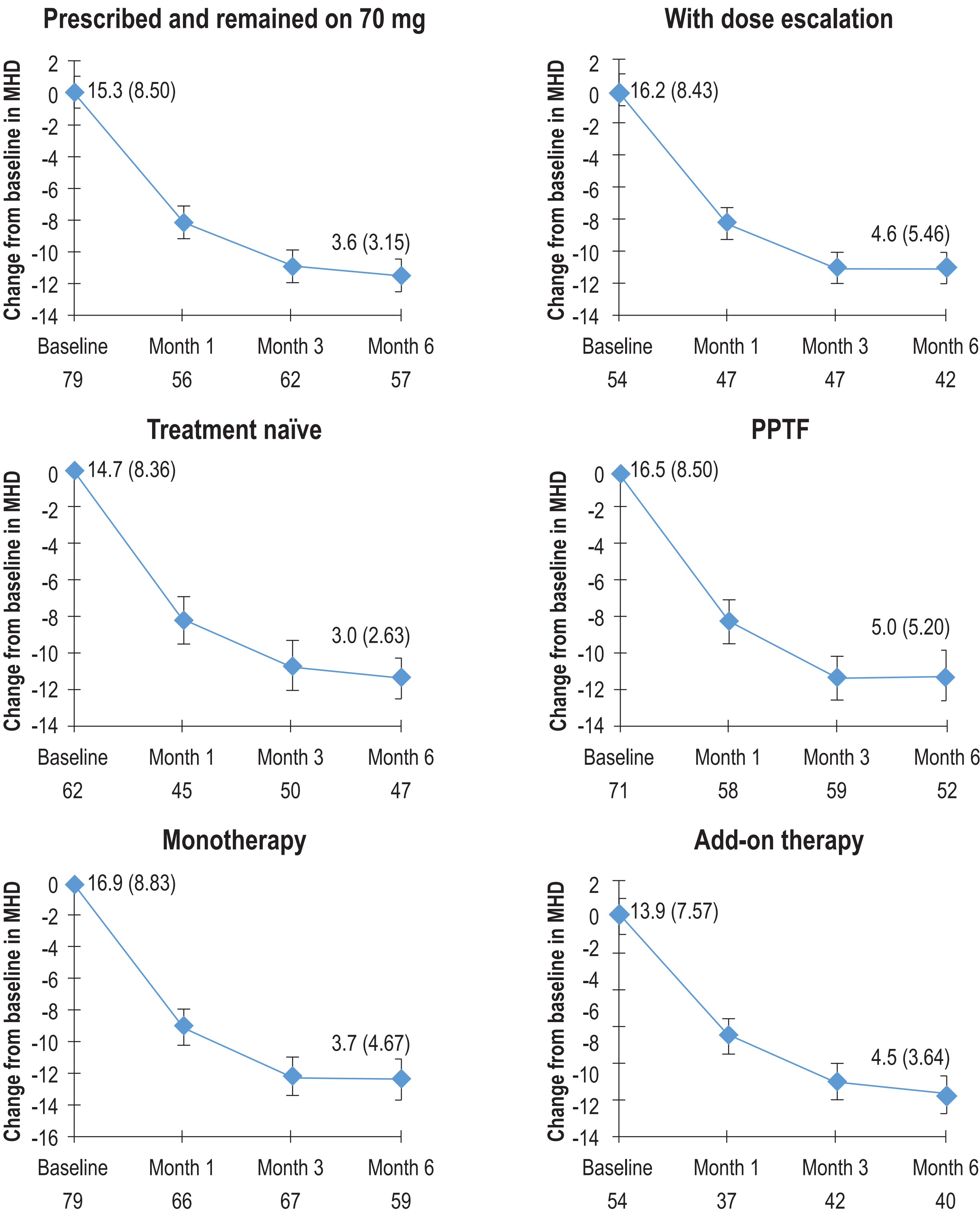

MHD, monthly headache/migraine days; PPTF, previous preventive treatment failure; SD, standard deviation

Mean ± standard error values are plotted and baseline mean MHD (SD) are provided for each plot.

Dose escalation: from 70 mg to 140 mg.

Baseline is defined as the last observation on the day of or before the first dose of the study drug.

After baseline, only patients with a value at both baseline and the respective month are included.

Fig. S3 Proportion of patients achieving <50%, 50% to <75%, 75% to <100% and 100% reduction in MHD over time – with and without dose escalation, by treatment naïve and failure and by monotherapy and add-on therapy

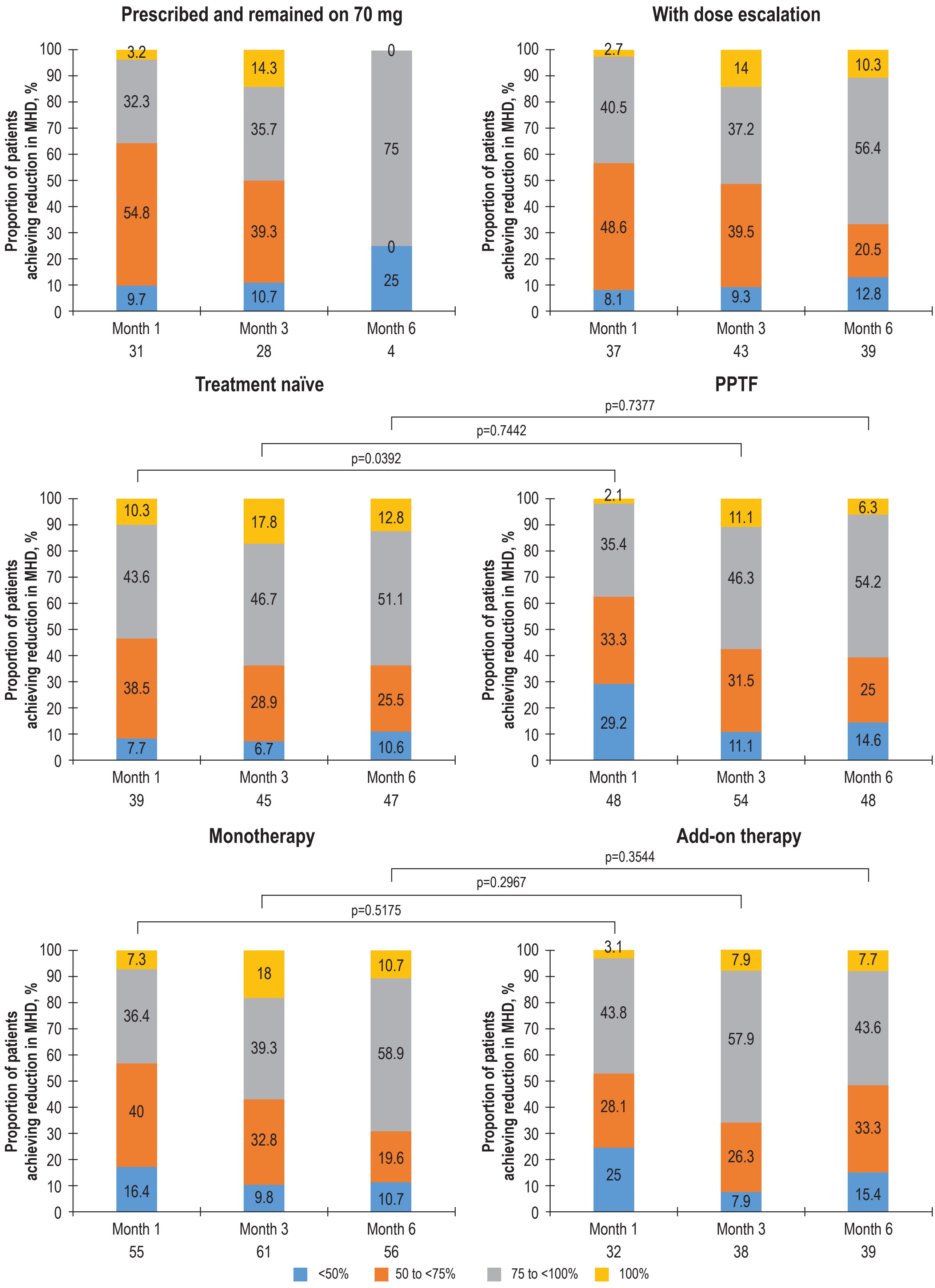

MHD, monthly headache/migraine days; PPTF, previous preventive treatment failure
Dose escalation: from 70 mg to 140 mg.
Percentages are based on the total number of evaluable subjects (n) at the respective visit in the full analysis set.

Fig. S4 Change from baseline in MSMD – with and without dose escalation, by treatment naïve and failure and by monotherapy and add-on therapy

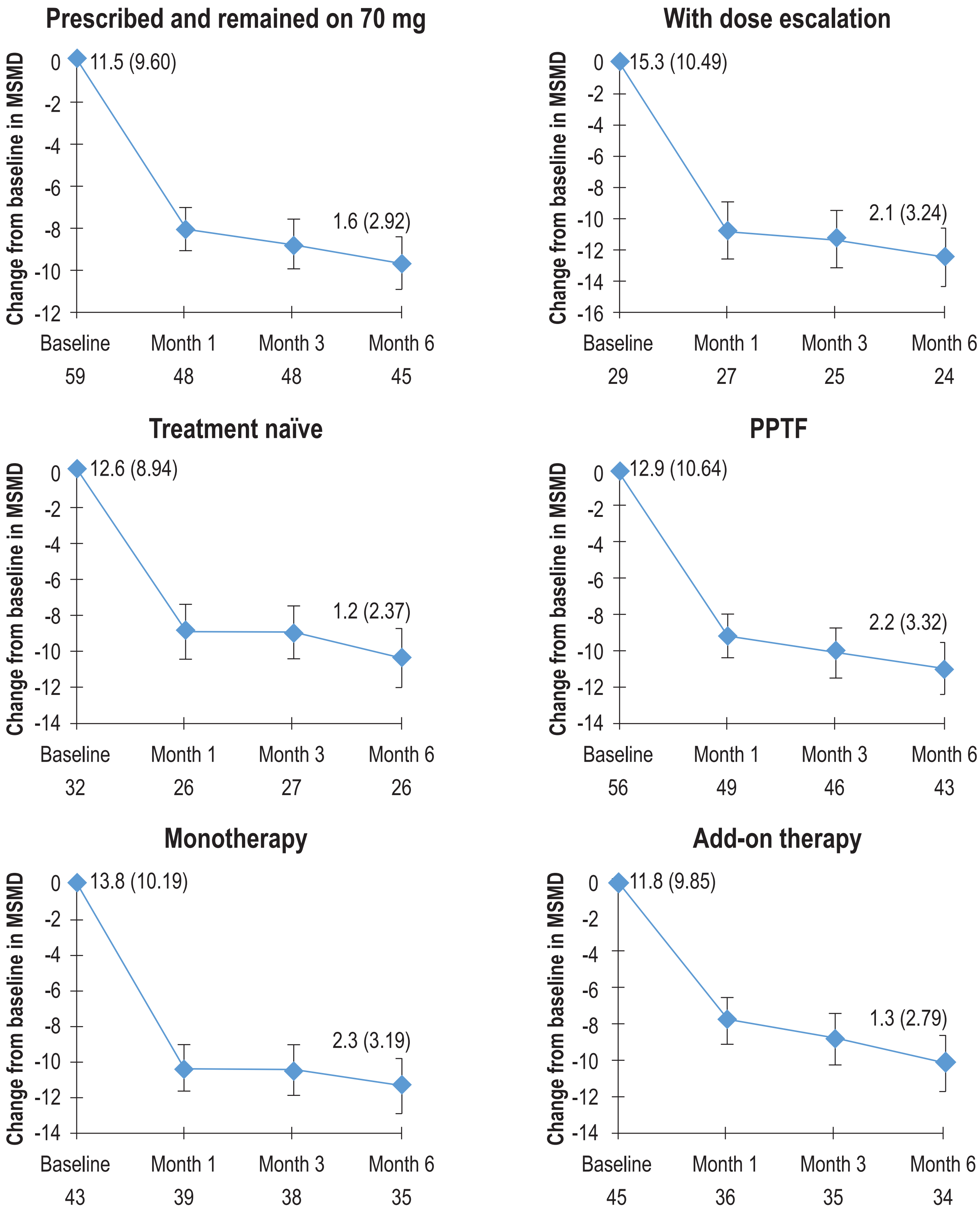

MSMD, monthly acute migraine-specific medication days; PPTF, previous preventive treatment failure; SD, standard deviation
Mean ± standard error values are plotted and baseline mean MSMD (SD) are provided for each plot.
Dose escalation: from 70 mg to 140 mg.
Baseline is defined as the last observation on the day of or before the first dose of the study drug.
After baseline, only patients with a value at both baseline and the respective month are included.

Fig. S5 Proportion of patients achieving <50%, 50% to <75%, 75% to <100% and 100% reduction in MSMD over time – with and without dose escalation, by treatment naïve and failure and by monotherapy and add-on therapy

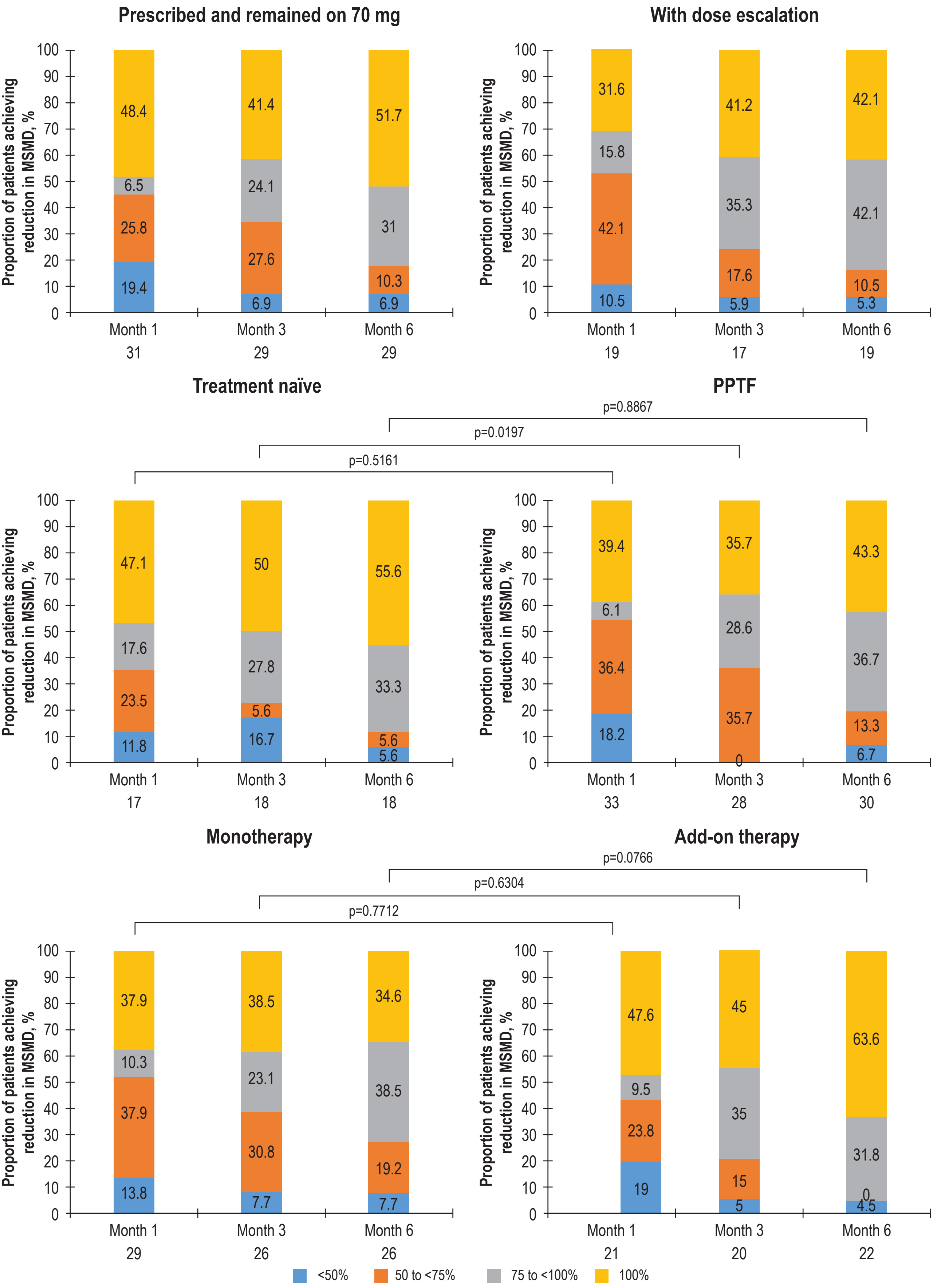

MSMD, monthly acute migraine-specific medication days; PPTF, previous preventive treatment failure

Dose escalation: from 70 mg to 140 mg.

Percentages are based on the total number of evaluable patients at the respective visit in the full analysis set.
